# [18F]FDG and [18F]FLT positron emission tomography imaging following treatment with belinostat in human ovary cancer xenografts in mice

**DOI:** 10.1186/1471-2407-13-168

**Published:** 2013-04-01

**Authors:** Mette Munk Jensen, Kamille Dumong Erichsen, Camilla Bardram Johnbeck, Fredrik Björkling, Jacob Madsen, Peter Buhl Jensen, Maxwell Sehested, Liselotte Højgaard, Andreas Kjær

**Affiliations:** 1 Cluster for Molecular Imaging, Faculty of Health and Medical Sciences, University of Copenhagen, Blegdamsvej 3B, 12.3.11, Copenhagen N, 2200, Denmark; 2 Department of Clinical Physiology, Nuclear Medicine & PET, Rigshospitalet, Denmark; 3 Topotarget A/S, Symbion Science Park, Fruebjergvej 3, Copenhagen, 2100, Denmark; 4 Present address: Department of Drug Design and Pharmacology, Faculty of Health and Medical Sciences, University of Copenhagen, Copenhagen, Denmark

**Keywords:** Positron emission tomography (PET), Belinostat, Histone deacetylase inhibitor, [18F]FLT, [18F]FDG, Computed tomography (CT)

## Abstract

**Background:**

Belinostat is a histone deacetylase inhibitor with anti-tumor effect in several pre-clinical tumor models and clinical trials. The aim of the study was to evaluate changes in cell proliferation and glucose uptake by use of 3’-deoxy-3’-[^18^F]fluorothymidine ([18F]FLT) and 2-deoxy-2-[^18^F]fluoro-D-glucose ([18F]FDG) positron emission tomography (PET) following treatment with belinostat in ovarian cancer *in vivo* models.

**Methods:**

*In vivo* uptake of [18F]FLT and [18F]FDG in human ovary cancer xenografts in mice (A2780) were studied after treatment with belinostat. Mice were divided in 2 groups receiving either belinostat (40 mg/kg ip twice daily Day 0–4 and 6–10) or vehicle. Baseline [18F]FLT or [18F]FDG scans were made before treatment (Day 0) and repeated at Day 3, 6 and 10. Tracer uptake was quantified using small animal PET/CT.

**Results:**

Tumors in the belinostat group had volumes that were 462 ± 62% (640 mm^3^) at Day 10 relative to baseline which was significantly different (P = 0.011) from the control group 769 ± 74% (926 mm^3^). [18F]FLT SUVmax increased from baseline to Day 10 (+30 ± 9%; P = 0.048) in the control group. No increase was observed in the treatment group. [18F]FDG SUVmean was significantly different in the treatment group compared to the control group (P = 0.0023) at Day 10. Within treatment groups [18F]FDG uptake and to a lesser extent [18F]FLT uptake at Day 3 were significantly correlated with tumor growth at Day 10.

**Conclusions:**

[18F]FDG uptake early following treatment initiation predicted tumor sizes at Day 10, suggesting that [18F]FDG may be a valuable biomarker for non-invasive assessment of anti-tumor activity of belinostat.

## Background

During development of new anti-cancer drugs methods to discriminate between effective and non-effective compounds and, on an individual patient basis, between responders and non-responders are of wide interests. For this purpose different imaging biomarkers are studied. The non-invasive imaging modality positron emission tomography (PET) assesses biological processes in intact living tissue. The tracer 3’-deoxy-3’-[^18^F]fluorothymidine ([18F]FLT) is a thymidine analogue that is used to image cell proliferation *in vivo* by PET, by measuring the activity of thymidine kinase 1 (TK1) which is up-regulated in the S-phase of cell cycle [1-6]. Pre-clinical studies have evaluated tumor cell proliferation by use of [18F]FLT PET after treatment with several different anti-cancer agents in different tumor models. The results are variable, ranging from a good correlation between early changes in [18F]FLT tumor uptake and tumor response to no change in [18F]FLT tumor uptake despite a good tumor response [7-17]. The FLT tracer has been validated against the proliferation marker Ki67 in several tumor types [18-20]. Ki67 protein measurements by immunohistochemistry are currently considered the gold standard for measurement of cell proliferation in tumor tissue specimens.

The tracer 2’-deoxy-2’-[^18^F]fluoro-D-glucose ([18F]FDG) is today the most widely used PET tracer for detecting and characterizing cancers. Changes in [18F]FDG uptake following anti-cancer treatment have been analyzed in several clinical studies; however, with variable results [21,22]. The Response Evaluation Criteria In Solid Tumors (RECIST) is a common method to assess tumor response by use of anatomical imaging techniques as computed tomography (CT) and magnetic resonance imaging (MRI) [23,24]. One disadvantage of using the tumor size as a response criterion for treatment is the amount of time it requires before a volume response becomes evident. Therefore new biological measurements are studied, and new guidelines have been suggested using e.g. [18F]FDG PET for measurement of treatment effect [25].

Belinostat (PXD101) is a histone deacetylase (HDAC) inhibitor, a relatively new class of anti-cancer drugs inhibiting the enzymes that deacetylate histone proteins. Histone acetylation is on the epigenetic level involved in regulation of gene expression. Belinostat induces anti-cancer activity in part by enhancing histone acetylation in tumor cells which causes alterations in gene expression [26-28]. However, the exact mechanism of how the aberrant gene expression causes anti-tumor activity remains unknown. Belinostat inhibits growth of human ovarian cancer cell lines *in vitro* and belinostat has anti-tumor activity *in vivo* in human A2780 ovarian cancer xenografts in mice [26,27]. The anti-tumor activity of belinostat is both related to inhibition of cell proliferation and induction of apoptosis and in several human cancer cell lines belinostat has been shown to cause cell cycle arrest in the G2/M phase [29-31]. We therefore speculated that belinostat treatment would reduce uptake of the cell proliferation tracer [18F]FLT.

Ovarian cancer is the most lethal of the gynecological cancers in women, and although many patients show an initial response to chemotherapy, numerous patients relapse with drug-resistant metastases [32]. Belinostat has both been tested as monotherapy and in combination with different chemotherapeutics in various clinical trials including trials containing ovarian cancer patients [33-39]. However, biomarkers for assessing tumor sensitivity and stratifying patients into responders and non-responders to HDAC inhibitors are currently lacking [40].

The aim of this study was to investigate if [18F]FLT and [18F]FDG PET can be used as non-invasive imaging biomarkers for monitoring of belinostat treatment. To do so, we analyzed [18F]FLT and [18F]FDG uptake in a human ovary cancer mouse model before and during treatment with belinostat. Tracer uptake was compared with Ki67, TK1 and glucose transporter 1 (GLUT1) gene expression.

## Methods

### Tumor model

Animal care and all experimental procedures were performed under the approval of the Danish Animal Welfare Council (2006/561-1124). Female NMRI (Naval Medical Research Institute) nude mice (8 weeks old) were acquired from Taconic Europe (Lille Skensved, Denmark) and allowed to acclimatize for one week in the animal facility before any intervention was initiated. The human ovarian carcinoma cell line A2780 (a gift from R. Ozols, Fox Chase Cancer Center Philadelphia, PA, January 2004) was used. For establishment of xenografts, 10^7^ cells in 100 μL medium mixed with 100 μL Matrixgel™ Basement Membrane Matrix (BD Biosciences, San Jose, CA, USA) were injected subcutaneously into the left and right flank respectively during anesthesia with 1:1 v/v mixture of Hypnorm® (Janssen Pharmaceutica, Beerse, Belgium) and Dormicum® (Roche, Basel, Switzerland). The cell line was tested free of mycoplasma. A2780 was cultured in RPMI (Roswell Park Memorial Institute) medium 1640 + GlutaMAX (Invitrogen, Carlsbad, CA, USA) supplemented with 10% fetal calf serum (Biological Industries, Israel) and 1% penicillin-streptomycin (Invitrogen) in 5% CO_2_ at 37°C.

### Synthesis of [18F]FLT and [18F]FDG

[18F]FLT was synthesized using 3-N-Boc-1-[5-O-(4,4'-dimethoxytrityl)-3-O-nosyl-2-deoxy-β-D-lyxofuranosyl]thymine as precursor on a GE TracerLab MX Synthesizer as previously described [41]. All reagents and [18F]FLT cassettes were purchased from ABX (Radeberg, Germany). [18F]FDG was acquired from daily productions at Rigshospitalet (Copenhagen, Denmark).

### Experimental design

*In vivo* uptake of [18F]FLT and [18F]FDG in human ovary cancer xenografts in mice (A2780) was studied at various time points after treatment initiation. When tumor volumes were approximately 100 mm^3^ mice were divided in 2 groups receiving either belinostat (40 mg/kg ip) or vehicle (L-arginine 80 mg/kg in isotonic sterile saline) twice daily Day 0–4 and Day 6–10. Baseline [18F]FLT or [18F]FDG PET scans were made before treatment and repeated at Day 3, 6 and 10 after treatment initiation. Tumor volume was followed by CT during the experiments [42]. Tumor volumes were calculated relative to volume at baseline. At Day 10 tumors were excised and gene expression of Ki67, TK1 and GLUT1 were analyzed by qPCR.

### microPET and microCT imaging

The mice were injected i.v. with 9.5 ± 0.2 (mean ± SD) MBq [18F]FLT or 10.0 ± 0.3 (mean ± SD) MBq [18F]FDG. Mice were fasted overnight before each [18F]FDG PET scan [43]. One hour after tracer injection mice were anaesthetized with 3% sevofluran (Abbott Scandinavia AB, Solna, Sweden) mixed with 35% O_2_ in N_2_ and fixed on a bed in presence of three fiducial markers allowing fusion of PET and CT pictures. A PET scan was acquired using a MicroPET Focus 120 (Siemens Medical Solutions, Malvern, PA, USA) followed by a microCT scan acquired with a MicroCAT® II system (Siemens Medical Solutions) as previously described [41]. PET data were arranged into sinograms and subsequently reconstructed with the maximum a posteriori (MAP) reconstruction algorithm. The pixel size was 0.866 × 0.866 × 0.796 mm and in the center field of view the resolution was 1.2 mm full-width-at-half-maximum.

PET and microCT images were fused in the Inveon software (Siemens Medical Solutions). Before fusion region of interests (ROIs) were drawn on the CT pictures manually by qualitative assessment covering the whole tumors and subsequently tumor volume and tracer uptake, assessed by standard uptake value (SUV) was generated by summation of voxels within the tomographic planes. SUV was calculated according to the formula (C_T_*W)/D_inj_, where C_T_ is tissue radioactivity concentration, W is weight of the animal and D_inj_ is injected dose. SUVmean was calculated from the mean radioactivity concentration and SUVmax was calculated from the voxel with the highest tracer concentration.

### Quantitative real-time polymerase chain reaction (qPCR)

Total RNA was isolated from the biopsies with TRI reagent® following the manufacturer’s instructions (Molecular Research Center Inc., OH, USA). The concentration of the RNA was determined by NanoDrop 1000 (Thermo Fisher Scientific, Wilmington, DE, USA). Total RNA (0.3 μg) was reversed transcribed using the Affinityscript™ QPCR cDNA Synthesis kit (Stratagene, La Jolla, CA, USA) according to the manufacturer’s instructions. Samples were cooled down and stored at −20°C until further use.

Primers were designed using Beacon Designer (PREMIER Biosoft, Palo Alto, CA, USA). Primer sequences were Ki67-FP: 5’-tcccgcctgttttctttctgac-3’, Ki67-RP: 5’-ctctccaaggatgatgatgctttac-3’, TK1-FP: 5’-gccgatgttctcaggaaaaagc-3’, TK1-RP: 5’-gcgagtgtctttggcatacttg-3’, GLUT1-FP: 5’-catcatcttcatcccggc-3’, GLUT1-RP: 5’-ctcctcgttgcggttgat-3’, GUSB-FP: 5’-tgagcaagactgatacca-3’, GUSB-RP: 5’-gctagaatagatgaccacaa-3’, HPRT-FP: 5’-caaagcctaagatgagagt-3’, HPRT-RP: 5’-gccacagaactagaacat-3’. For each gene the optimal primer concentration was found. All assays were optimized to have efficiencies between 95% and 105%. All samples were run in triplicate using one μl of cDNA. To each sample a no-reverse transcription control (NoRT) was included, and on each plate a no-template control (NTC) was included.

Gene expression was quantified on a Mx3000P® real-time PCR system from Stratagene. All gene of interests (GOIs) and reference genes were quantified with Brilliant® SYBR® Green QPCR Master Mix (Stratagene). The following thermal profile was used in all experiments: 10 minutes of denaturation at 95°C followed by 45 cycles of 30 seconds denaturation at 95°C, 1 minute of annealing at 60°C and 1 minute extension at 72°C. A dissociation curve was afterward acquired by denaturation of the products for 1 minute at 95°C followed by a stepwise increase in temperature from 55°C to 95°C with steps of 0.5°C/cycle where the duration of each cycle was 18 seconds.

QPCR data were analyzed in the qBase program. The relative quantification of the GOIs was calculated using two reference genes [44]. Data are presented as fold changes in the treatment compared to the control group at Day 10. The level of the GOIs was normalized to the geometric mean of two reference genes. The two most stable reference genes were found from a panel of 12 candidates in the human reference gene panel (TATAA Biocenter AB, Göteborg, Sweden) by use of the geNorm algorithm.

### Statistical analysis

Comparison between the treatment and control group was calculated using unpaired student’s *t*-test. Paired *t*-test was used for intra-group comparisons. Bonferroni correction of P-values for multiple comparisons was applied. Correlations between SUVmean or SUVmax and tumor growth were calculated using linear regression. Calculations were made in PASW 18.0 (IBM Corporation, Armonk, New York, USA). Data are reported as mean ± SEM unless stated otherwise and p < 0.05 was considered statistically significant.

## Results

### Tumor volume

**Figure 1:**
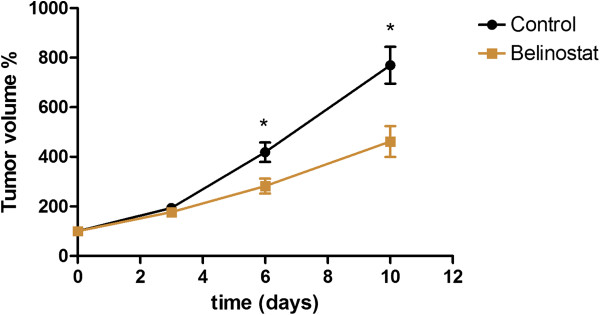
***Effect of belinostat on the growth of A2780 xenografts. ***Tumor volume was determined by microCT. The mice were treated with belinostat (40 mg/kg ip twice daily Day 0–4 and 6–10) or vehicle. N = 12-13 tumors/group. *) p < 0.05 treatment versus control group at same time point.

Tumors in the control group had volumes that were 419 ± 39% at Day 6 and 769 ± 74% at Day 10 relative to baseline. In the belinostat group tumors were 282 ± 30% at Day 6 and 462 ± 62% at Day 10 relative to baseline which were significantly less than the control group both at Day 6 (p = 0.029) and Day 10 (p = 0.011) (Figure 1). At baseline the tumor sizes in the treatment (130 ± 23 mm^3^) and control group (118 ± 19 mm^3^) were identical.

### [18F]FDG and [18F]FLT microPET imaging

**Figure 2:**
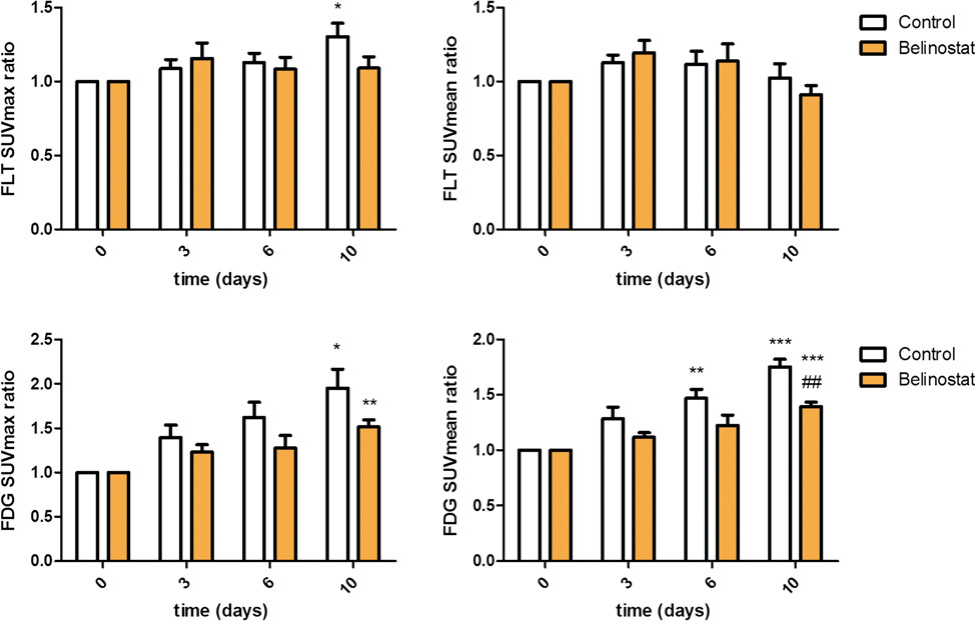
***Tumor uptake of [18F]FLT and [18F]FDG following treatment with belinostat. ***Mice with human ovary cancer xenograft tumors were treated with either belinostat (40 mg/kg ip twice daily Day 0–4 and 6–10) or vehicle and they were subjected to PET imaging with either [18F]FLT or [18F]FDG at baseline and Day 3, Day 6 and Day 10 after treatment start . [18F]FLT (upper panel) and [18F]FDG (lower panel) uptake measured as SUVmax relative to baseline (left panel) and SUVmean relative to baseline (right panel). N = 5-7 tumors/group. *) p < 0.05, **) p < 0.01, ***) p < 0.001 versus baseline in same treatment group. #) p < 0.05, ##) p < 0.01, ###) p < 0.001 treatment versus control group at same time point.

**Figure 3:**
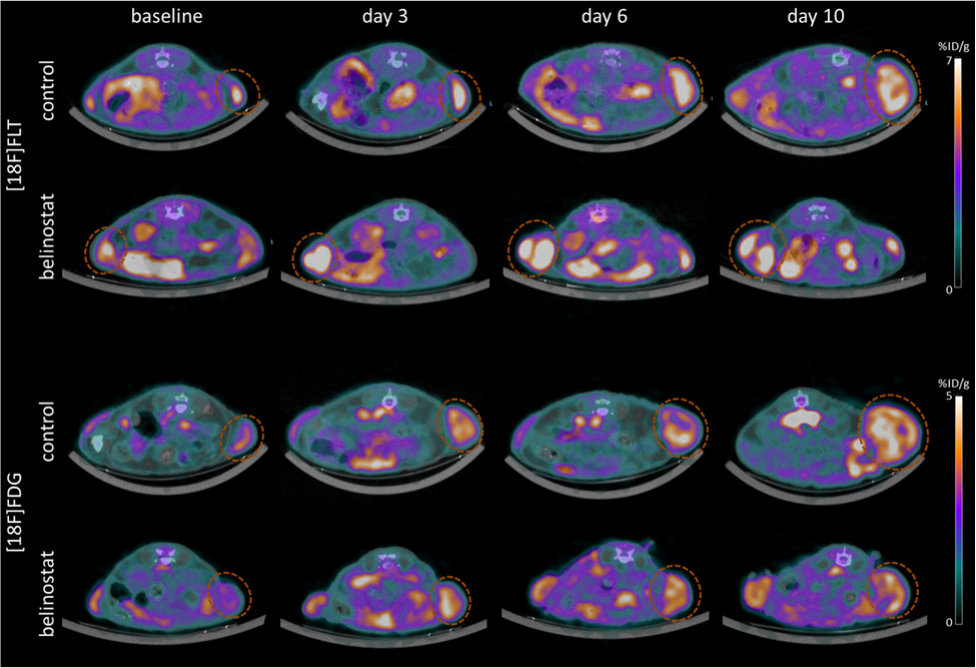
***PET/CT images. ***Representative combined PET/CT images of [18F]FLT scans (upper panel) and [18F]FDG scans (lower panel) of mice treated with belinostat (40 mg/kg ip twice daily Day 0–4 and 6–10) or vehicle respectively. Tracer uptake was measured in the same animals at baseline, Day 3, Day 6 and Day 10. Dotted circles indicate the tumors.

When studied in treatment groups, no differences in [18F]FLT uptake between treatment and control groups were observed at any time points for either SUVmean or SUVmax. [18F]FLT SUVmax uptake increased from baseline to Day 10 (+30 ± 9%; p = 0.048) in the control group. No increase in [18F]FLT SUVmax was observed in the treatment group at Day 10 (Figures 2, 3).

**Figure 4:**
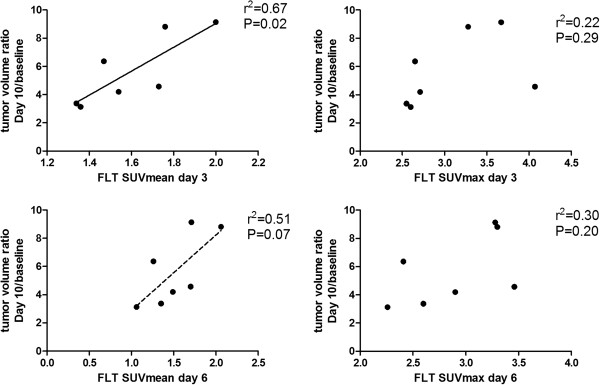
***Correlations between tumor uptake of [18F]FLT and tumor growth. ***Tumor growth was measured as tumor volume ratio Day 10/baseline. The graphs show tumor growth correlated with uptake of [18F]FLT SUVmean and SUVmax at Day 3 and Day 6 respectively.

[18F]FDG SUVmax was increased at Day 10 compared to baseline in both the control (+95 ± 22%; p = 0.035) and treatment group (+52 ± 8%; p = 0.0015). No significant difference in SUVmax between treatment and control group was observed at any time point. [18F]FDG SUVmean was significant different in the treatment compared to the control group (p = 0.0023) at Day 10. Compared to baseline, [18F]FDG SUVmean uptake was increased at Day 6 (+47 ± 8%; p = 0.013) and Day 10 (+75 ± 7%; p = 0.0013) in the control group. Compared to baseline, [18F]FDG SUVmean uptake was increased at Day 10 (+40 ± 4%; p < 0.001) in the treatment group (Figures 2, 3). Within treatment groups [18F]FLT SUVmean Day 3 was significantly correlated with relative tumor volume Day 10/baseline in the belinostat group (r^2^ = 0.67; p = 0.02). [18F]FLT SUVmean Day 6 was correlated with tumor growth Day 10/baseline in the belinostat group (r^2^ = 0.51; p = 0.07) however not significant (Figure 4).

**Figure 5:**
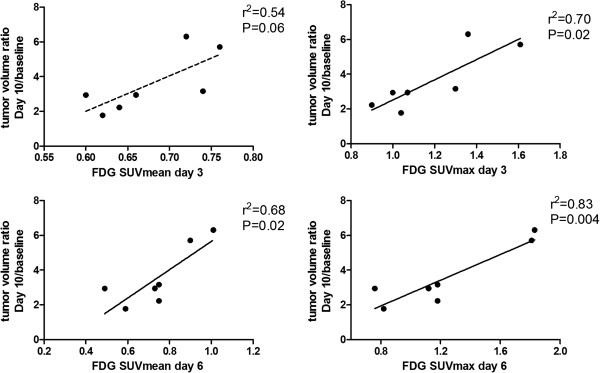
***Correlations between tumor uptake of [18F]FDG and tumor growth. ***Tumor growth was measured as tumor volume ratio Day 10/baseline. The graphs show tumor growth correlated with uptake of [18F]FDG SUVmean and SUVmax at Day 3 and Day 6 respectively.

Within treatment groups [18F]FDG SUVmean Day 3 was correlated with tumor growth Day 10/baseline in the belinostat group (r^2^ = 0.54; p = 0.06), however not significant. [18F]FDG SUVmean Day 6 was significantly correlated with tumor growth Day 10/baseline (r^2^ = 0.68; p = 0.02). [18F]FDG SUVmax Day 3 was significantly correlated with tumor growth Day10/baseline (r^2^ = 0.70; p = 0.02) as were [18F]FDG SUVmax Day 6 (r^2^ = 0.83; p = 0.004) (Figure 5).

### Ki67, TK1 and GLUT1 gene expression

**Figure 6:**
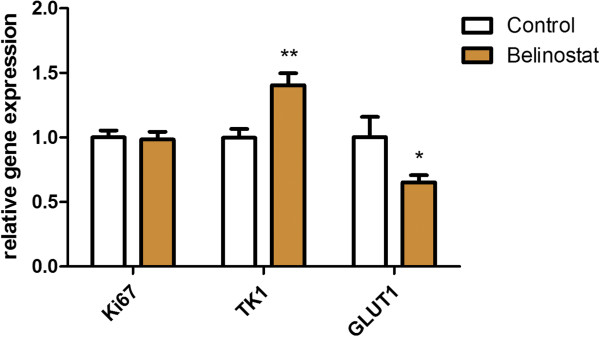
***Ki67, TK1 and GLUT1 gene expression.*** At Day 10 after treatment start all tumors were excised and total RNA was isolated and revers transcribed to cDNA. Expression of Ki67, TK1 and GLUT1 were measured with qPCR and normalized to the geometric mean of two reference genes. Data are presents as fold changes in the treatment compared to the control group at Day 10. N = 5-7 tumors/group. *) p < 0.05, **) p < 0.01, ***) p < 0.001 treatment versus control group at same time point.

The two most stable reference genes were beta-glucuronidase (GUSB) and hypoxanthine phosphoribosyltransferase 1 (HPRT). The levels of Ki67, TK1 and GLUT1 were normalized to the geometric mean of these two reference genes. The gene expression was measured at Day 10 in the treatment relative to the control group. Ki67 gene expression was unchanged in the treatment compared to the control group at Day 10. TK1 gene expression was higher in the treatment compared to the control group at Day 10 (1.40 ± 0.09 vs 1.00 ± 0.07; p = 0.006). GLUT1 gene expression was lower in the treatment group compared to the control group at Day 10 (0.65 ± 0.06 vs 1.00 ± 0.16; p = 0.05) (Figure 6).

## Discussion

In this study we found that [18F]FDG uptake following initiation of treatment with the HDAC inhibitor belinostat predicted tumor sizes at the end of treatment in a mouse model of human ovary cancer. We observed minor effects on [18F]FLT uptake following treatment with belinostat. In a previous study lower tumor uptake of [18F]FLT was observed following treatment with the HDAC inhibitor LAQ824 in a human colon carcinoma mouse model [11]. LAQ824 is, like belinostat, a hydroxamate HDAC inhibitor [45]. However, despite belonging to the same class of HDAC inhibitors, we did not find the same change in [18F]FLT uptake following treatment initiation with belinostat. The changes in [18F]FLT uptake was followed by a reduction in TK1 transcription and translation in the study with LAQ824 [11]. Interestingly, we observed an increase in TK1 gene expression following treatment with belinostat. It has been shown in a colon cancer cell line, that treatment with belinostat reduces the levels of thymidylate synthase (TS) [28]. An effect of TS inhibition can be up-regulation of the salvage nucleotide pathway leading to increased uptake of thymidine and hence [18F]FLT [8,46,47]. This could be an explanation for the increase in TK1 that we observe at Day 10 following treatment with belinostat. Despite the increase in TK1 gene expression no increase in [18F]FLT uptake was observed at Day 10. The connection between TK1 gene expression and TK1 protein expression was not analyzed in this study so further analysis are needed in order to elucidate whether the observed increase in gene expression actually translate into increased protein expression and activity and how it correlates with [18F]FLT uptake. That belinostat prevented increase in [18F]FLT uptake in human ovary cancer xenografts is in line with one study were the [18F]FLT uptake was analyzed following treatment with belinostat in a mouse model of human colon cancer [48]. Effective treatment with belinostat prevented increase in [18F]FLT uptake in the colon cancer model.

Even though we did not find a decrease in [18F]FLT uptake in the belinostat group, within the treatment group [18F]FLT SUVmean at Day 3 and 6 was correlated with tumor growth at Day 10. The tumors having the lowest uptake of [18F]FLT at Day 3 and 6 following initiation of treatment with belinostat were those in which the treatment was most effective.

Previously we have observed that [18F]FLT uptake was reduced after initiation of effective anti-cancer therapy in the A2780 tumor model [41,49]. Thus, thymidine requirement in the A2780 tumor model is most likely dependent on the salvage pathway. Other studies have also observed changes in [18F]FLT uptake after initiation of effective anti-cancer therapies in other models of human ovarian cancer. In a pre-clinical study [18F]FLT uptake was decreased following effective mTOR inhibition with everolimus in a pre-clinical cisplatin-resistant ovarian tumor model [50]. In cisplatin-sensitive ovarian cancer xenografts both [18F]FLT and [18F]FDG uptake were decreased day 4 after initiation of treatment with cisplatin [51].

Compared to the [18F]FLT data, we observed a higher influence on [18F]FDG uptake following treatment with belinostat. At Day 10 uptake of [18F]FDG was decreased in the treatment group compared to the control group. The difference at day 10 did only reach significant difference for SUVmean and not for SUVmax. SUVmean is the mean tracer concentration in tumor and SUVmax is a measure of the pixel within the tumor which has the highest tracer concentration. An explanation to the non-significant change in SUVmax despite changes in SUVmean could therefore be because the anti-cancer treatment is less effective and does not inhibit glucose uptake in the most aggressive parts of the tumor and therefore no significant difference between SUVmax for the treatment and control group was observed. Another explanation to the differences could be that the difference for SUVmax did not reach statistical significance due to a type II error because of the limited amount of animals included in the study.

The difference in [18F]FDG uptake between the treatment and control group was supported by underlying changes in gene expression of GLUT1. At Day 10 GLUT1 expressions were lower in the treatment compared to the control group. Other HDAC inhibitors likewise decrease GLUT1 gene expression [52]. Glucose transporters accounts for [18F]FDG transport into cancer cells and GLUT1 expression has in many studies been positively correlated with [18F]FDG uptake [53,54].

Within the treatment group the level of [18F]FDG uptake at Day 3 and 6 was correlated with treatment effect at the end of the study. The tumors which had the lowest [18F]FDG uptake at Day 3 and 6 following treatment start were the tumors which responded best to the treatment. Projecting this into a clinical situation will allow identification of the patients responding best to the treatment. Advantage of this information can be taken in two ways. Firstly, selection of which patients to be included in a clinical trial can be determined depending on drug sensitivity determined early in the treatment course. This will make identification of new compounds which are effective in only a small subset of patients easier. Secondly, in clinical practice, treatment modifications in non-responding patients during a treatment course may be undertaken.

Some of the main limitations of the present study were the lack of protein expression levels of molecular markers in tumor tissue. It is therefore unknown whether or not the gene expression levels of Ki67, TK1 and GLUT1 reflected the protein levels of the matching proteins. However, in other studies a positive correlation between Ki67 protein and gene expression has been observed [55,56]. Furthermore does the present study not describe whether the early changes in tracer uptake will be predictive for long-term growth inhibition of the pre-clinical ovary cancer model and if the data acquired in this pre-clinical mouse model can be translated to clinical studies.

No regression in tumor volume was observed following treatment with belinostat; however, the tumor growth was lower in the treatment compared to the control group, thus confirming the anti-cancer effect of belinostat. It is known, that the belinostat compound exerts tumor stasis rather than tumor shrinkage [26-28]. Identification of effect with drugs exerting tumor stasis can be difficult, as the conventional anatomical imaging modalities CT and MRI measure treatment effect by assessing changes in tumor size. A tumor stasis effect of the anti-cancer treatment can consequently be missed by these anatomical imaging modules. Therefore, identification of biological biomarkers is of great value in treatment regimes involving tumoristatic compounds [40].

## Conclusions

In conclusion, we found that [18F]FDG uptake early following treatment initiation with belinostat predicted tumor sizes at Day 10, suggesting that [18F]FDG PET may be a biomarker for non-invasive assessment of anti-tumor activity of belinostat. The results from this study supports the addition of [18F]FDG PET scans during clinical trials with belinostat where it may also be used for selection of subjects that may enter such studies.

## Abbreviations

[18F]FLT: 3’-deoxy-3’-[^18^F]fluorothymidine; [18F]FDG: 2-deoxy-2-[^18^F]fluoro-D-glucose; PET: Positron emission tomography; CT: Computed tomography; TK1: Thymidine kinase 1; HDAC: Histone deacetylase; ROIs: Region of interests; SUV: Standard uptake value; GOI: Gene of interests; TS: Thymidylate synthease

## Competing interests

The following co-authors have conflict of interests: Peter Buhl Jensen: Ownership Interests and Employment in Topotarget A/S. Maxwell Sehested: Ownership Interests and Employment in Topotarget A/S. Fredrik Björkling: Employment in Topotarget A/S. Kamille Dumong Erichsen: Employment in Topotarget A/S. All other authors have no conflict of interests.

## Authors’ contributions

Conceived and designed the experiments: MMJ KDE FB PBJ LH MS AK. Performed the experiments: MMJ KDE CBJ JM. Analyzed the data: MMJ KDE AK. Wrote the paper: MMJ AK. Revised and approved the paper: KDE CBJ FB JM PBJ LH MS AK. All authors read and approved the final manuscript.

## Pre-publication history

The pre-publication history for this paper can be accessed here:

http://www.biomedcentral.com/1471-2407/13/168/prepub
